# Comparison of the outcomes of cannulated screws vs. modified tension band wiring fixation techniques in the management of mildly displaced patellar fractures

**DOI:** 10.1186/s12891-015-0719-7

**Published:** 2015-10-06

**Authors:** Tao Lin, Junbin Liu, Baojun Xiao, Dehao Fu, Shuhua Yang

**Affiliations:** Department of Orthopedics, Qilu Hospital of Shandong University, Jinan, Shandong Province China; Department of Orthopedics, Union Hospital, Tongji Medical College, Huazhong University of Science and Technology, Wuhan, Hubei Province China; Department of Traumatic Surgery, Jining No. 1 Peoples Hospital, Jining, Shandong China

**Keywords:** Patellar fractures, Cannulated screws, Lysholm scores, Modified tension band

## Abstract

**Background:**

K wire fixation with tension band wiring has conventionally been used for the open reduction and internal fixation of the patella. However, it suffers from distinct disadvantages such as implant irritation, need for open reduction, incidence of palpable implants, and need for subsequent implant removal. A smaller incision with percutaneous fixation may be an alternative to this established conventional technique. Thus, the purpose of this trial was to compare the treatment outcomes of patients with mildly displaced patellar fractures treated with closed reduction and percutaneous cannulated screw fixation (CRCF) as compared to open reduction and tension band wiring fixation (ORTF). Specifically, we aimed to determine whether cannulated screw fixation was associated with improved clinical outcomes at 12 months as measured using the Lysholm score, pain scores, degree of flexion, range of motion, time to radiographic union, radiographic outcomes, and complication rates.

**Methods:**

Sixty-three patients with transverse patellar fractures displaced less than 8 mm were included in this prospective, randomized, controlled trial, with 52 patients in the final data analysis. Thirty-two patients were operatively treated by CRCF with either two or three cannulated screws. Thirty-one patients were operatively treated by conventional ORTF using the modified tension band technique. At postoperative intervals of 3, 6, and 12 months, knee function was evaluated using the Lysholm score, pain was assessed using the visual analog scale (VAS) score, and active knee extensions and flexion were measured in degrees by goniometry.

**Results:**

The CRCF group had average Lysholm scores of 84.4 ± 5.8, 86.7 ± 6.4, and 93.2 ± 5.3 after 3, 6, and 12 months, respectively, which were significantly greater than those of the ORTF group (79.0 ± 5.3, *p* = 0.001; 81.5 ± 4.6, *p* = 0.002; and 89.8 ± 6.2, *p* = 0.039, respectively). Lower pain and squatting scores were the main reasons for the poorer Lysholm scores in the ORTF group. The VAS scores showed that the CRCF group had lower pain scores and better flexion and total range of motion (ROM) compared with the ORTF group after 3 and 6 months, although both groups had similar outcomes after 12 months. The mean fracture healing time of 2.65 months was similar in the CRCF groups (2.77 months; *p* = 0.440). Complication rates were 3/26 (11.5 %) in the CRCF group and 14/26 (53.4 %) in the ORTF group. Two patients in the CRCF group and eight patients in the ORTF group experienced skin irritation. In addition, two (7.7 %) patients in the CRCF group and 11 (42.3 %) patients in the ORTF group required implant removal because of symptoms due to the presence of the implants.

**Conclusion:**

Surgical treatment of mild displaced (less than 8 mm) transverse patellar fractures by the CRCF technique provides satisfactory clinical results and excellent knee function, with little pain and a low incidence of complications at early follow-up (up to 6 months). These results suggest that the CRCF technique may be a superior alternative to conventional ORTF. Registration Trial (Chinese Clinical Trial Register): Current Controlled Trials ChiCTR-PRCH-14005017, registration dates 2014-06-14.

## Background

Patella injuries constitute up to 2 % of all fractures, with 70–90 % of these having a transverse fracture pattern [1–4]. Conventionally, open reduction and internal fixation is indicated for transverse patellar fractures with displacement of greater than 3 mm, articular incongruity of 2 mm or more, or a disrupted extensor mechanism [2, 5–7]. The tension band wiring technique is the most common method of transverse patella fracture fixation [1, 2, 5, 8–10]. This technique, although used widely, has certain distinct disadvantages. These include the requirement for a long skin incision with a substantial soft tissue dissection to provide adequate visualization of the fracture as well as the joint surfaces [5, 6, 11, 12], the potential for damage to the blood supply to the patella [7, 10, 13, 14], the need for subsequent prolonged rehabilitation [15–17], as well as greater blood loss, increased surgical time, and the inherent risk associated with prolonged anesthesia [10, 17, 18]. On the other hand, postoperative complications are also common with this method, especially implant-related issues, with a reported incidence as high as 22–53 % of cases [15, 19]. The issues include wire breakage and migration, postoperative loss of reduction (22–45 % of cases) [15, 19], and a high rate of symptomatic implant irritation (8–48 %) [15–17], with rates of subsequent implant removal for symptomatic hardware ranging from 10–55 % [15, 17, 19]. In view of the problems and complications associated with conventional K wire fixation and tension band wiring, several alternative approaches have been described. These include closed reduction and fracture fixation using inter-fragmentary screws or cannulated screws with or without supplementary tension band wiring through the screw. Additionally, some authors have used arthroscopy to visualize and facilitate the accurate reduction of the articular surfaces [3, 7, 9, 10, 14–17]. The advantage of these techniques is the minimally invasive nature of the procedure, resulting in minimal soft tissue disruption and a shorter rehabilitation period [7, 14, 17, 18]. Closed reduction and stable fixation ensures that the blood supply to the patella is not disturbed [10, 14, 16]. Although there are no clear guidelines, it is believed that fractures that are displaced by more than 8 mm are likely to be accompanied by disruption of the extensor mechanism [10]. Based on these observations, it has been generally felt that fractures with less than 8 mm displacement are the only ones that are amenable to the closed reduction and fixation procedures [3, 10, 14, 15]. Currently, there is a lack of comparative research of percutaneous cannulated screw fixation and open reduction and fixation using a modified tension band. Thus, we felt that a prospective, randomized, controlled, clinical trial would help to evaluate the relative merits and disadvantages of these two different methods of fixation.

The purpose of this exercise-based prospective randomized trial was to compare closed reduction and percutaneous fixation with cannulated screws (CRCF) versus open reduction and internal fixation with tension band wiring (ORTF) of transverse patellar fractures. The study aimed to determine whether cannulated screw fixation was associated with improved clinical outcomes at 12 months as measured using the Lysholm score, pain scores, knee flexion, knee range of motion, time to radiographic union, better radiographic outcomes, and complication rates.

## Methods

### Patients and methods

This study was carried out over a period of 5 years between June 2008 and June 2013 in the Department of Orthopedics, Union Hospital of Tongji Medical College, Huazhong University of Science and Technology. Patients with open fractures or multiple fractures, who presented more than 5 days after the initial injury, who had type C1.2 or C1.3 fractures, or who had a transverse patellar fracture with displacement greater than 8 mm according to the computed tomography (CT) scan were excluded from the study. Before undertaking this trial, a pilot study of 14 patients was done to ensure feasibility. Taking into account the possible Lysholm scores and the expected extension, flexion and range of motion (ROM) at 12-month follow-up, we found that the difference between the averages of the two groups was the smallest at 12-month follow-up, with the CRCF group having an average Lysholm score of 93.7 ± 5.7, which was better when compared to the ORTF group (90.3 ± 6.9, *p* = 0.042). Using a single-tail design with α = 0.05 and β = 0.10, as well as the standard deviation (5.9) and the difference of the Lysholm scores (3.4) from the pilot study, we determined that the total target sample size should be at least 52 patients (26 patients per treatment).

A total of 63 patients were recruited and enrolled in the study, based on our initial planned budget. Patients were assigned to one of two treatment groups following simple randomization procedures (computerized random numbers). 32 patients underwent CRCF under the assistance of fluoroscopy, while the other 31 patients were treated by ORTF. At postoperative intervals of 3, 6, and 12 months, knee function was evaluated using the Lysholm score, pain assessment using the visual analog scale (VAS) score, and active knee extension, and flexion were measured in degrees by goniometry.

**Fig. 1:**
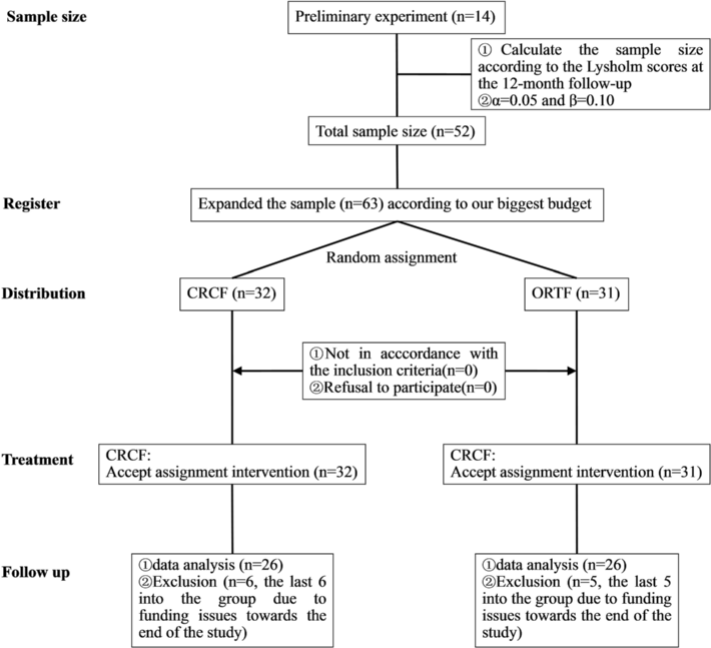
The CONSORT format flow chart

Subsequently, the last 11 patients to be recruited (CRCF: 6, ORTF: 5) were excluded from the data analysis as they were only followed up for 3 months due to lack of research funding. The rest 52 patients were followed up for a minimum of 12 months as a part of this study, with no loss of follow-up (Fig. 1).

X-rays were evaluated to determine initial fracture type, postoperative reduction, implant position, and fracture union. CT scans were obtained pre-operationally by all study candidates to accurately evaluate the patellar fracture patterns, displacement magnitude of the fracture classification, and also to exclude transverse fractures of the patella that had comminution at the fracture site. All transverse patellar fractures with displacement less than 8 mm were prospectively enrolled in this study. Apart from confirming inclusion in the study, the CT scan also helped in preoperative planning and served as an intraoperative reference.

X-ray assessments were performed on the standard lateral views of the affected knee joint. These views were obtained with the affected knee joint positioned on the bed surface, keeping the X-ray tube film at a 90-cm distance. Both the internal and external condyle of the femur overlapped, and the magnification rate was 10 %. All the data was repeatedly measured three times by one person.

This was an expertise-based randomized trial, with one surgeon performing all cannulated screw procedures and the second performing the tension band technique.

### Demographics

The demographics of both groups are shown in Table 1. There were no statistically significant differences between the two groups, including age, side, gender, fracture classification, mechanism, fracture displacement, and operative time to injury. The average age of the 52 patients was 51.7 years old (range, 22–76 years old). The most common fracture type was classified as a transverse, middle third patellar fracture (AO/OTA 45-C1.1), which occurred in 61.5 % of patients. The major mechanism of injury was a low-energy fall in both groups. The average preoperative displacement between the two fragments was 4.3 ± 1.8 mm in the CRPF group versus 4.7 ± 1.7 mm in the ORIF group. The operation was performed at a mean of 3 days after the initial injury.

**Table 1:** Demographics of the two groups

Demographic	CRCF group (*n* = 26)	ORTF group (*n* = 26)	*p* value
Gender			0.578
Male	15	13	
Female	11	13	
Age (years)	50.8 ± 16.3	52.5 ± 17.4	0.725
Side			0.266
Left	16	12	
Right	10	14	
Mechanism			0.774
Fall	15	14	
Sports-related trauma	4	6	
Traffic accident	7	6	
AO/OTA type			
Transverse, middle (45-C1.1)	15 (57.7 %)	17 (65.4 %)	0.841
Transverse, proximal (45-C1.2)	4 (15.4 %)	3 (11.5 %)	
Transverse, distal (45-C1.3)	7 (27.0 %)	6 (23.0 %)	
Interfragmentary gap (mm)	4.3 ± 1.8	4.7 ± 1.7	0.471
Operative time to injury (days)	2.8 ± 1.2	3.3 ± 1.4	0.184

*CRCF* closed reduction and percutaneous cannulated screw fixation, *ORTF* open reduction and modified tension band fixation

### Preoperative assessment

On initial presentation in the emergency room, radiographic examination of the knee including both anteroposterior and lateral views of the knee were obtained. The fracture pattern was confirmed after clinical assessment and cross-sectional radiography (CT). The Orthopedic Trauma Association (OTA) system was used to classify each fracture. The preoperative displacement between the two fragments was evaluated by a CT scan. The affected knee was immobilized in extension to avoid further displacement of the fracture.

### Operative procedure

#### Closed reduction and percutaneous fixation with cannulated screws

**Fig. 2:**
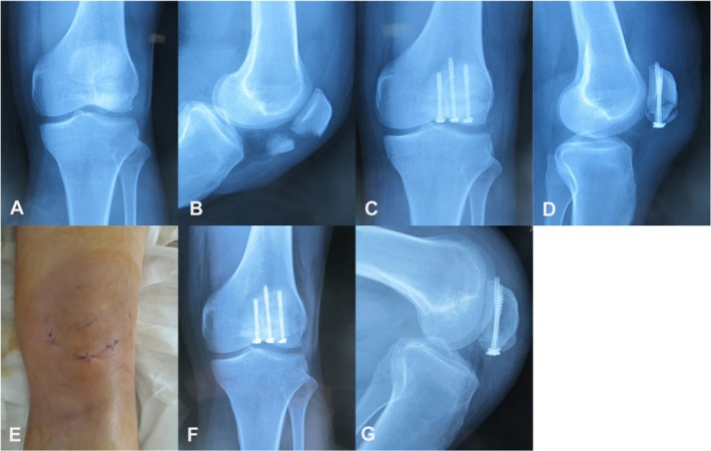
Closed reduction and percutaneous cannulated screw fixation. **a** and **b**, Preoperative radiograph of a 45-year-old man who had a mildly displaced transverse patellafracture. **c** and **d**, Postoperative radiograph showing the proper placement of the three screws. **e** The minimally invasive approach shows three small incisions less than 8 mm with minimal soft tissue disruption. **f** and **g**, The 8-week postoperative radiograph showingthat the patella fracture has union with agood degree of knee flexion

One dose of preoperative antibiotics (Ceftriaxone) was given to all patients within 30 min before induction of the anesthesia, and another postoperative dose was given 12 h later. Under spinal anesthesia, the patients were placed in the supine position on a standard radiolucent operating table with the knee in full extension. Fine adjustments of the reduction were carried out by manipulating the fragments under fluoroscopic guidance. Two pointed reduction clamps were applied percutaneously at the far end of each fragment with a 5-mm stab incision at both the median and lateral side of the proximal and distal one-fifth of the patella (Fig. 2e). Thereafter, the knee was then flexed to 15–25° to facilitate insertion of the guide wires. The reduction was rechecked by fluoroscopy. Two Kirschner wires (K-wires, 1.5-mm diameter) were percutaneously drilled longitudinally, perpendicular to the fracture line, from the proximal or distal pole of the patella through a stab incision. These two parallel guide wires were separated by 2 cm in the cornal plane. The fracture fragments were fixed over the two guide wires using 4.0-mm cannulated screws (Sanatmetal, Hungary, titanium alloy, partially threaded, diameter of 4.0 mm, and length of 35–60 mm). The length of the screw used was determined by the total length of the guide wire minus the length exposed outside both poles of the patella. The exact length of the screw for insertion was measured to ensure that the screw tip penetrated the cortex of the superior patellar pole or the inferior patellar pole and that the heads of the screws were buried into the distal cortical bone. In all patients, washers were used to enhance interfragmentary compression as the screw was advanced across the fracture site. The stability of the reduction and fixation was checked by flexing and extending the knee. A third cannulated screw was added when the stability was not satisfactory, or when osteopenia or osteoporosis was identified (Fig. 2).

#### Open reduction and internal fixation with tension band wires

**Fig. 3:**
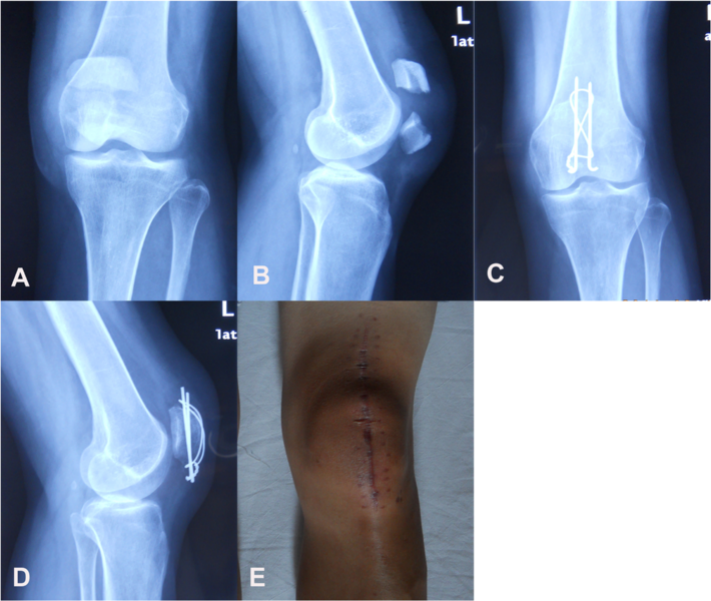
Open reduction and fixation using a modified tension band. **a** and **b**, Preoperative radiograph of a 48-year-old woman who had a transverse patella fracture. **c** and **d**, Postoperative radiograph showing the proper placement of wire and Kirschner wire. **e** The knee joint anterior midline approach showing that a long skin incision of approximately 14 cm was required in this case

The ORTF technique was performed using a previously described standard procedure for open reduction and fixation with a figure-eight tension band wire technique (Fig. 3) [1, 20].

#### Postoperative treatment and rehabilitation

External immobilizers, e.g., plaster casts or long knee immobilizers, were not used in any of the patients. Patients performed quadricep-femur contraction exercises soon after the operation. Unrestricted passive ROM was started postoperatively at the earliest time point, depending on the patient’s pain tolerance. Active ROM was encouraged by 3 weeks postoperatively, and full weightbearing was started by 8 weeks [3, 15]. The main outcome measurements included Lysholm score, VAS score, flexion, ROM, complications, reoperations, and radiographic union.

#### Postoperative follow-up and assessment

All patients were followed up biweekly in the first month both clinically and with conventional radiographs (including lateral and AP patellar radiographs). Patients were reexamined at 3, 6, and 12 months after the operation, and evaluated on clinical as well as radiological parameters. Union of the fracture was defined when more than 80 % bridging of the fracture line by bone was observed on the lateral side of the radiograph [3]. The final results of the procedure were assessed using the Lysholm knee scoring scale [21].

Post-operative displacement was defined as a fracture site displacement of greater than 2 mm as compared to the position on the immediate postoperative imaging tests. We defined a mild infection as a superficial infection that did not involve the bone, joint, or implants and that was successfully treated on an outpatient basis with the use of oral antibiotics. The implant, or the hardware, was termed as ‘painful hardware’ when the skin was irritated by prominent hardware or migration of the K-wires was seen. Tension band loosening or migration was defined as radiographic disengagement from the K-wires, which can impact fracture stability. In all cases, hardware removal was indicated when there was painful hardware, skin irritation, or post-operative fracture displacement before healing.

### Ethics and consent

This study was performed in compliance with the Helsinki Declaration and has been granted approval from the Ethics Committee of Union Hospital of Tongji Medical College. Written informed consent was obtained from all patients enrolled in this study, and all patients were over 18 years old. Consent to publish Figs. 2 and 3 and the information contained in the figure legends were also obtained from each patient.

### Statistical methods

A total of 52 subjects were included in this analysis. We used SPSS 18 statistical software for Windows for all analyses. For comparison of the normally distributed variables, a two-tailed Student’s t-test was used; for non-normal distributions, the Mann–Whitney U test was used. Differences in proportions for categorical variables were assessed with the use of chi-squared tests. A Fisher exact test was used for categorical comparisons when the expected cell frequency was <10. The P value was set at a significance level of 0.05.

## Results

### Clinical results

At postoperative intervals of 3, 6, and 12 months, the CRCF group had average Lysholm scores of 84.4 ± 5.8, 86.7 ± 6.4, and 93.2 ± 5.3, respectively, which were significantly greater than those in the ORTF group (79.0 ± 5.3, *p* = 0.001; 81.5 ± 4.6, *p* = 0.002; and 89.8 ± 6.2, *p* = 0.039) (Table 2).

**Table 2:** Results of the two groups after each follow-up

	CRCF (*n* = 26)	ORTF (*n* = 26)	*P* value
Pain (VAS)			
3	2.6 ± 2.1	4.5 ± 2.7	0.006
6	1.4 ± 2.1	3.2 ± 2.8	0.013
12	0.5 ± 1.1	1.1 ± 1.9	0.160
Flexion (°)			
3	109.0 ± 10.5	100.2 ± 12.0	0.007
6	138.3 ± 10.9	127.7 ± 16.6	0.009
12	140.4 ± 10.3	136.9 ± 11.0	0.246
ROM (°)			
3	104.8 ± 13.8	93.9 ± 15.1	0.009
6	136.7 ± 12.7	125.2 ± 18.5	0.012
12	139.2 ± 11.8	135.0 ± 12.7	0.219
Lysholm score			
3	84.4 ± 5.8	79.0 ± 5.3	0.001
6	86.7 ± 6.4	81.5 ± 4.6	0.002
12	93.2 ± 5.3	89.8 ± 6.2	0.039

*CRCF* closed reduction and percutaneous cannulated screwfixation, *ORTF* open reduction and modified tension band fixation, *VAS* visual analog scale, *ROM* range of motion

After each follow-up period, analysis of the Lysholm score subgroups revealed that a lower mean difference of pain (25 points) and a lower mean difference of squatting scores (5 points) were the main reasons for the poorer Lysholm scores in the ORTF group. There were no differences in Lysholm scores in different groups based on the initial fracture type (*p* = 0.447). Moreover, there were no differences in the Lysholm scores when patients were stratified by age (*p* = 0.903) or by gender (*p* = 0.800).

The visual analog scale (VAS) scores were significantly lower in the CRCF group at 3 and 6 months (*p* = 0.003 and *p* = 0.013), with scores in the CRCF group of 2.6 ± 2.1 and 1.4 ± 2.1, and scores in the ORTF group of 4.5 ± 2.7 and 3.2 ± 2.8, respectively. However, the VAS scores were similar between the two groups at the final follow-up.

Compared with the ORTF group, the CRCF group had gained significant flexion and total ROM after 3 and 6 months, but the mean values were similar at 12 months (Table 2). The CRCF group had average flexion values of 109.0 ± 10.5°, 138.3 ± 10.9°, and 140.4 ± 10.3°, when compared to those of the ORTF group (100.2 ± 12.0°, *p* = 0.007; 127.7 ± 16.6°, *p* = 0.009; and 136.9 ± 11.0°, *p* = 0.246, respectively). The CRCF group had average total ROM values of 104.8 ± 13.8°, 136.7 ± 12.7°, and 139.2 ± 11.8°, respectively, which were better than those of the ORTF group (93.9 ± 15.1°, *p* = 0.009; 125.2 ± 18.5°, *p* = 0.012; and 135.0 ± 12.7°, *p* = 0.0219).

### Radiological results

Radiographic union occurred at a mean of 11 weeks post-surgery in both groups. The mean fracture healing time for the CRCF group was 2.65 months (range, 2–4 months), which was similar to that seen in the ORTF group (2.77 months) (*p* = 0.44) (Table 3). In the CRCF group, 18 patients were treated with two cannulated screws, and 8 patients received a three screw configuration fixation. There were no significant differences in the rate of fracture healing based on the number of screws, although three cannulated screw fixation needed an additional 12 min of intraoperative fluoroscopy. The radiographic results at the 12-month follow-up showed that the interfragmentary gap attained with the cannulated screw technique compared favorably with the tension band figure-eight wiring technique. There was one patient in the CRCF group and two patients in the ORTF group in whom a fracture postoperative displacement of 2 mm or greater occurred.

**Table 3:** Results of the two groups at the 12-month follow-up

	CRCF (*n* = 26)	ORTF (*n* = 26)	*P* value
Fracture healing time (months)	2.65 ± 0.61	2.81 ± 0.8	0.440
Postoperative interfragmentary gap			0.934
0 mm	19 (73.1 %)	20 (77.0 %)	
≤ 2 mm	5 (19.2 %)	4 (15.4 %)	
≥ 3 mm	2 (7.7 %)	2 (7.7 %)	
Implant removal	2	11	
Pain (VAS)	0.5 ± 1.1	1.1 ± 1.9	0.160
Extension (°)	1.2 ± 1.7	1.9 ± 1.9	0.133
Flexion (°)	140.4 ± 10.3	136.9 ± 11.0	0.246
ROM (°)	139.2 ± 11.8	135.0 ± 12.7	0.219
Lysholm score	93.2 ± 5.3	89.8 ± 6.2	0.039

*CRCF* closed reduction and percutaneous cannulated screw fixation, *ORTF* open reduction and modified tension band fixation, *VAS* visual analog scale, *ROM* range of motion

### Complications

The complication rates were 3/26 (11.5 %) in the CRCF group and 14/26 (53.4 %) in the ORTF group (Table 4). Fracture displacement of 2 mm or more occurred in one patient in the CRCF group at 2 weeks and two patients in the ORTF group immediately following surgery. The patient in the CRCF group then underwent a second operation, which was performed directly by the ORTF technique after the fracture displacement was discovered. The two displaced fractures in the ORTF group healed with the protection of a brace, and the internal fixation was removed 12 months later.

**Table 4:** Complications after 12 months

	CRCF (*n* = 26)	ORTF (*n* = 26)
Displaced fragment	1 (3.8 %)	2 (7.7 %)
Infection	0	2 (7.7 %)
Painful hardware	2 (7.7 %)	8 (30.8 %)
Tension band loosening or migration	0	3 (11.5 %)
Implant removal	2 (7.7 %)	11 (42.3 %)
Reoperation rate	1 (3.8 %)	11 (42.3 %)

*CRCF* closed reduction and percutaneous cannulated screwfixation, *ORTF* open reduction and modified tension band fixation

The overall incidence of postoperative infection was 3.8 % (*n* = 2). Two patients (7.7 %) in the ORTF group had a postoperative infection as compared with none in the CRCF group. Both of the infections were classified as mild as they were superficial infections that did not involve the bone, joint, or implant; the patients were successfully treated on an outpatient basis with the use of antibiotics.

There were two patients in the CRCF group and eight patients in the ORTF group who experienced skin irritation as a consequence of prominent hardware. After 12 months, there were 2 (7.7 %) patients in the CRCF group and 11 (42.3 %) patients in the ORTF group who underwent removal of the symptomatic hardware.

## Discussion

The results of our study showed that the CRCF was associated with significantly better clinical outcomes up to 12 months as measured using the Lysholm score. However, while this technique was associated with favorable pain scores, flexion, and ROM at early follow-ups (up to 6 months), there were no differences in these measures at the final 12-month follow-up. Furthermore, there was no difference in the mean time to union between groups.

In this study, patients managed using the CRCF technique achieved better knee function, especially in the early postoperative period. The reported advantages of the percutaneous fixation technique include avoidance of extended incisions, preservation of the blood supply to the patella [10], and the possibility of a simpler removal of all hardware in the clinical setting [22]. In this study, we found that the percutaneous fixation technique was associated with less pain, potentially facilitating early knee motion and preventing muscular atrophy and intra-articular adhesions due to immobilization. It has also been demonstrated that early motion has some advantages for joint cartilage perfusion and nutrition [10].

The minimally invasive technique of percutaneous fixation with cannulated screws has the advantages of causing less destruction of the soft tissues and less implant irritation to the soft tissues, reducing the risk of postoperative wound complications and encouraging early rehabilitation. Generally, the CRCF technique was performed through 6–7 stab incisions of 5 ± 2 mm, while a longer skin incision was required in the ORTF group to provide adequate visualization of the fracture as well as the joint surface [5, 11, 12, 22]. Consequently, the ORTF technique potentially jeopardizes the blood supply of the fragments and the biological environment at the fracture site required for optimal bone regeneration and healing [7, 13, 14]. In this group, two patients had delayed wound healing with mild infections, possibly due to the open nature of the procedure. Additionally, substantial soft tissue dissection can result in postoperative stiffness and delayed rehabilitation [10, 17, 18], potentially decreasing both ROM and functional outcome; however, the results of this study suggest that the difference in ROM does not persist after 6 months.

The CRCF technique, given its minimally invasive nature, can provide stable fixation and at the same time permit early mobilization and accelerated rehabilitation. Previously conducted biomechanical investigations have revealed that the cancellous screws alone or in combination with tension band wiring provide stable fixation provided that the fracture is well reduced with less than a 1-mm gap between the fracture fragments [23]. As such, Benjamin et al. have recommended screw fixation for transverse patellar fractures in patients with adequate bone stock [24]. Similarly, studies by Dargel et al. and Carpenter et al. have demonstrated that, in certain cases, interfragmentary screw fixation provides fixation that is even more stable and rigid than the modified tension band wiring technique [25, 26]. Authors have revealed that there are several benefits of percutaneous fixation of patellar fracture with the assistance of fluoroscopy or arthroscopy [3, 7, 9, 10, 14–17], thus providing satisfactory reduction and rigid fixation [3, 7, 9, 10, 14, 15, 17].

Post-operative displacement was defined as a fracture site displacement of greater than 2 mm as compared to the position on the immediate postoperative imaging tests. In our study, there were 6 out of 26 patients in each group with a postoperative interfragmentary gap, and displacement occurred at 2 weeks in one patient in the CRCF group (45-C1.3). It was believed to be not a fixation failure secondary to an inadequate screw or due to appropriate placement. Rather, we suspected that the extremely poor bone quality and unlimited and aggressive early active mobilization contributed to the resultant failure. The cannulated screws could not achieve the optimal direct interfragmentary compression force and failed in this patient, but there was not enough evidence to support that the cannulated screws were associated with a greater risk of failure in all 45-C1.3 fractures. Two failures in the ORTF group were attributed to technical errors associated with the tension band immediate post operation. We hypothesized that the soft tissue interposition between the tension band and the superior pole of the patella and inadequate wire tension of the figure-eight wire onto the patellar surface were the main causes.

Biomechanical studies have suggested that cannulated screws can provide a reduction and a direct interfragmentary compression force, while the tension band wiring technique cannot achieve direct fracture site compression and can only transform distraction forces into compression forces [1, 5]. Moreover, the flexibility of K-wires can counteract a part of the force that should act on the fractured bone [5]. In order to function properly, the patella must be able to withstand significant axial forces [8]. Especially under full knee extension, a tension wire system may not provide compression at the fracture and may even lead to further fracture displacement [8], which may be aggravated by the concomitant presence of soft tissue atrophy and loss of muscle tone. According to Baydar et al. [27], cannulated screws are more durable against distraction forces than the modified tension band technique to treat transverse patellar fractures. Similar studies have reported that fractures stabilized with a modified tension band displace significantly more than those fixed with screws alone or screws plus a tension band in simulated knee extensions (*p* < 0.05) [28].

An advantage of cannulated screws is the potential for percutaneous application. Currently, several treatment options have been introduced for displaced transverse patellar fractures, including cannulated screws [10, 14] as well as cannulated screws with a tension band wire or a titanium cable placed through a screw [1, 3, 18, 29]. A disadvantage of the percutaneous fixation method includes its technical difficulty [16]. For example, it has been noted that it can be difficult to place K-wires appropriately with a minimally invasive technique, and it may have a substantial learning curve [26]. In addition, burying the knot when using the percutaneous fixation method with tension band wiring may also be a difficult task [2, 9]. Finally, technical errors in tension-band wire placement or wire twists have been identified as the cause of fixation failure with the use of percutaneous techniques [18, 19].

CRCF had a markedly lower complication rate of 11.5 % when compared to 53.4 % for ORTF. Other clinical reports have similarly shown that osteosynthesis of closed, displaced, multi-type patellar fractures with open techniques can offer acceptable results with a fixation failure rate of 3.5–22 % [18, 19]. In our study, the prevalence of a middle transverse fracture without disruption of the extensor mechanism (45-C1.1) was 61.5 %; and the rate of fixation failure occurring with fracture displacement greater than 2 mm was low, with 7.7 % in the CRCF group and 3.8 % in the ORTF group.

Painful hardware was the most common complication in the ORTF group, which occurred in 30.1 % of patients, and tension band loosening and migration was the second major complication, seen in 11.5 % of patients. Although symptomatic implant irritation is not as serious a complication as fixation failure or postoperative infection, it has the potential to impede rehabilitation, potentially leading to stiffness that may require a second intervention or manipulation under anesthesia with additional hospitalization and cost. The rates of implant removal for symptomatic hardware have been reported to range from 37 to 55 %, although no large study has directly compared the rate of implant removal for specific fixation constructs [15, 18].

In our study, the reoperation rate was 11.5 % (4/26) for the CRCF group, which was significantly less than 42.3 % (11/26) in the ORTF group. The main reason is that fixation with K-wires is associated with a high rate of hardware removal compared with screws. Other reports have shown that cannulated screws with a low profile caused less soft-tissue irritation than K-wires [10, 18].

In the ORTF group, reduction of the joint surface was verified with both direct visualization and radiographic assessment; however, the surgical exposure needed may have resulted in serious injury to the soft tissue. In the CRCF group, anatomical reduction of the articular surface was checked by repeated fluoroscopy only; however, there was no post-traumatic arthritis as a result of malreduction or incongruous articular surface seen at the 12-month follow-up. Nevertheless, a longer follow up on determining the post traumatic arthritis incidence may be required. The arthroscopic technique is a safe and reproducible method for transverse patellar fractures and may allow direct visualization of the joint surface [3]. However, it is not indicated for major separation displaced fractures with disruption of the extensor mechanism [3, 10, 14]. There may be several advantages of arthroscopy in the treatment of patellar fractures: removal or wash out of small chondral or osteochondral fragments, visualization of the articular surface for reduction [14, 15], evaluation of the articular surface, and assurance that the screw does not intrude upon the patellar cartilage [15].

Reduction by indirect means is not difficult during surgery, especially for fractures with less than 8 mm displacement. These fractures usually have an intact extensor mechanism and are thus amenable to the closed reduction and fixation procedures. We have commented that cannulated screws are technically difficult to place properly because a screw that is too short will not provide adequate fixation, while a long screw may skin irritation, causing it to fail prematurely. But the cannulated screws should not be redrilled and replaced more than two times to avoid compromising the strength of interfragmentary compression. Occasionally, when one or two screws were thought to be in a suboptimal position, a third screw was placed, depending on the size of the fragment. Eighteen patients received two cannulated screw fixation, and only eight patients were operatively treated with three cannulated screws. There were no significant differences in fracture healing between these groups; however, three cannulated screw fixation needed an extra 12 min of intraoperative fluoroscopy time.

The extensor mechanism of the knee is an intertwined net of tendinous, capsular, and ligamentous tissues. The patella is located between the quadriceps and patellar tendons, which are involved in the extensor movement mechanism of the knee joint. The patella functions as the link between the quadriceps and patellar tendon and functions by increasing the leverage of the quadriceps tendon and also lifting the extensor mechanism from the rotational center of the knee. The majority of transverse patellar fractures are a result of excessive tensile force across the extensor mechanism and thus represent a disruption of the extensor mechanism. The competence of the extensor mechanism post reconstruction is thus vital to ensure an optimal outcome and patient satisfaction. Active straight leg raising is the simplest and one of the most reliable ways to assess the integrity and function of the extensor mechanism.

Thus, one of the most important findings to be assessed on clinical evaluation is the loss of active extension of the knee or an inability to maintain the passively extended knee against gravity. But physical examination may be difficult, particularly if the injured knee has a severe hemarthrosis. Thus, only a small proportion of patients can usually perform this test on the first clinical examination. Once the pain was relieved, a repeat assessment was done and occasionally a secondary loss of reduction position was found in some patients. There is relevant documentary evidence to support that fractures that are displaced more than 8 mm are likely to be accompanied by disruption of the extensor mechanism, and some patients may be able to perform a straight leg raise, even in the presence of an extensor mechanism disruption, due to an intact iliotibial band and medial or lateral patellar retinaculum. But there was no quadriceps atrophy or weakness in either group at the 12-month follow-up, incomplete terminal extension of the knee, or subjective patient dissatisfaction with overall knee function.

In most of the literature examples, conservative treatment is limited to fractures with less than 2 mm of separation and no significant displacement of the articular surface. The management options for fractures with diastasis greater than 2 mm or articular step off are either a closed or an open reduction with supplemental fixation. It has been generally felt that fractures with less than 8 mm displacement are the only ones that are amenable to the closed reduction and fixation procedures [3, 10, 14, 15]. Although there are no clear guidelines, fractures that are displaced by less than 8 mm are likely to be accompanied with an intact extensor mechanism [10]. Thus, they are suitable for conservative treatment, especially when the patients have contraindications to operative intervention either because of medical reasons or because of refusal to consent for the procedure.

There were several limitations of this study. First, our technique was indicated for displaced transverse patellar fractures but is not recommended for transverse fragments displaced more than 8 mm. Second, this was a single-center and open-label trial, and the sample size was relatively small. Thus, this study design might be a cause of bias. Moreover, one experienced surgeon performed the CRCF technique, and another experienced surgeon performed the ORTF technique. Third, some researchers may feel that the follow-up period of one year is too short to reach an appropriate conclusion.

## Conclusion

The surgical treatment of transverse patellar fractures with less than 8 mm displacement by closed reduction with the percutaneous cannulated screw fixation technique was shown to provide satisfactory clinical results and excellent knee functions, with less pain and a low incidence of complications. This procedure could be a new option for the treatment of transverse patellar fractures.

## Abbreviations

CRCF: Closed reduction and percutaneous fixation with cannulated screws
ORTF: Open reduction and internal fixation with tension band wires
ROM: Total range of motion
